# A Local Desiccant Antimicrobial Agent as an Alternative to Adjunctive Antibiotics in the Treatment of Periodontitis: A Narrative Review

**DOI:** 10.3390/antibiotics12030456

**Published:** 2023-02-24

**Authors:** Iulia C. Micu, Alexandrina Muntean, Alexandra Roman, Ștefan I. Stratul, Emöke Pall, Andreea Ciurea, Andrada Soancă, Marius Negucioiu, Lucian Barbu Tudoran, Ada G. Delean

**Affiliations:** 1 Department of Periodontology, Faculty of Dental Medicine, Iuliu Haţieganu University of Medicine and Pharmacy Cluj-Napoca, Victor Babeş St., No. 15, 400012 Cluj-Napoca, Romania; 2 Department of Pedodontics, Faculty of Dental Medicine, Iuliu Hațieganu University of Medicine and Pharmacy Cluj-Napoca, Avram Iancu St., No. 31, 400117 Cluj-Napoca, Romania; 3 Department of Periodontology, Anton Sculean Research Center of Periodontal and Peri-Implant Diseases, Faculty of Dental Medicine, Victor Babeș University of Medicine and Pharmacy Timișoara, Bulevardul Revoluției din 1989, No.9, 300230 Timișoara, Romania; 4 Department of Infectious Disease, Faculty of Veterinary Medicine, University of Agricultural Sciences and Veterinary Medicine, Mănăștur St., No. 3-5, 400372 Cluj-Napoca, Romania; 5 Department of Prosthodontics, Faculty of Dental Medicine, Iuliu Haţieganu University of Medicine and Pharmacy Cluj-Napoca, Clinicilor St., No. 32, 400006 Cluj-Napoca, Romania; 6 Electron Microscopy Center, Department of Molecular Biology and Biotechnologies, Faculty of Biology and Geology, Babeş-Bolyai University, Clinicilor St., No. 5-7, 400006 Cluj-Napoca, Romania; 7 Electron Microscopy Integrated Laboratory (LIME), National Institute for Research and Development of Isotopic and Molecular Technologies, Institutul Național de Cercetare-Dezvoltare pentru Tehnologii Izotopice și Moleculare, 67-103 Donath St., 400293 Cluj-Napoca, Romania; 8 Department of Cariology, Endodontics and Oral Pathology, “Iuliu Hațieganu” University of Medicine and Pharmacy, Moților St., No. 33, 400001 Cluj-Napoca, Romania

**Keywords:** periodontitis, peri-implantitis, biofilms, antibacterial agents, HybenX

## Abstract

Periodontitis is one of the most common oral polymicrobial infectious diseases induced by the complex interplay between the altered subgingival microbiota and the host’s dysregulated immune-inflammatory response, leading to the initiation of progressive and irreversible destruction of the periodontal tissues and eventually to tooth loss. The main goal of cause-related periodontal therapy is to eliminate the dysbiotic subgingival biofilm in order to arrest local inflammation and further periodontal tissue breakdown. Because, in some cases, subgingival mechanical instrumentation has limited efficiency in achieving those goals, various adjunctive therapies, mainly systemic and locally delivered antimicrobials, have been proposed to augment its effectiveness. However, most adjunctive antimicrobials carry side effects; therefore, their administration should be precociously considered. HybenX^®^ (HY) is a commercial therapeutical agent with decontamination properties, which has been studied for its effects in treating various oral pathological conditions, including periodontitis. This review covers the current evidence regarding the treatment outcomes and limitations of conventional periodontal therapies and provides information based on the available experimental and clinical studies related to the HY mechanism of action and effects following its use associated with subgingival instrumentation and other types of dental treatments.

## 1. Introduction

Periodontitis is one of the most common chronic infectious-inflammatory diseases in humans and a major public health concern [1,2] that causes, in the absence of treatment, progressive and irreversible destruction of the tooth-supporting apparatus [3] and ultimately leads to edentulism and major oral functional impairments, and carries considerable negative consequences on the general health and overall quality of life of the affected patients [3,4,5].

The subgingival mechanical instrumentation is the gold standard of periodontitis treatment and determines a significant shift in the subgingival microbiota [6,7] as well as important clinical improvements [3,7], but sometimes has a limited efficiency in eliminating subgingival deposits and periodontal pockets [8]. Different adjunctive therapeutical strategies have been suggested to improve the effectiveness of subgingival mechanical instrumentation, especially in severe and high-risk cases [3,9], but no ideal or generally applicable product or technique can be recommended [3]. Although some locally delivered antimicrobials used in conjunction with subgingival mechanical instrumentation have been shown to determine a significantly greater reduction of periodontal pockets as compared to subgingival instrumentation alone, the differences have no real clinical significance (weighted mean difference (WMD) less than 0.5 mm). Hence, further investigations are needed to identify new efficient antimicrobial agents and products for locally delivered therapies in periodontitis cases [10,11].

One interesting commercial product, with antimicrobial and desiccation properties, based on a mixture of sulfonic/sulfuric acids, under the name of HybenX^®^ (HY) (Epien Medical, St. Paul, MN, USA) [12] has been proposed as a possible adjunctive approach to combat periodontal and peri-implant infections. However, the effects of HY were not reviewed in the recent periodontal treatment guideline [3]. Moreover, there is inconsistent information on its clinical benefits in periodontitis treatment and scarce data on its biocompatibility [13,14]. HY was initially used to treat oral ulcerative lesions due to its denaturation and antimicrobial properties [15]. Furthermore, its biofilm matrix disintegrating capacity, which enhances disinfection, made HY appealing for endodontic therapy [16].

The present paper summarizes current information on the periodontitis-associated microbiota and covers the available evidence related to the goals and limitations of the current periodontal treatment approaches and of the adjunctive therapies associated with the subgingival mechanical instrumentation. In addition, this paper explores the characteristics of HY that can aid biofilm removal, the product’s complex mechanisms of action, and the clinical periodontal outcomes following its use to provide essential arguments for a larger implementation of HY in clinical practice as an alternative to conventional adjunctive antibiotics.

## 2. Features of the Periodontitis-Inducing Dysbiotic Biofilm

Periodontitis onset is triggered by a complex makeup of bacteria forming the subgingival dysbiotic biofilm [17]. Periodontitis pathogenesis is currently supported by polymicrobial synergy and dysbiosis theories, according to which an increasing abundance of a synergistic polymicrobial community generates subgingival microbial dysbiosis and immune disruption in susceptible individuals [18,19,20].

Periodontitis-associated subgingival biofilm is characterized by an augmentation of newly dominant species, composed mainly of anaerobic genera from the phyla *Firmicutes*, *Proteobacteria*, *Spirochaetes, Bacteroidetes*, and *Synergistetes* rather than the colonization of new exogenous pathogenic species, which were previously considered not to belong to the health-related subgingival biofilms [21]. The current paradigm considers the periodontitis-associated polymicrobial community to be present in a very low abundance in periodontal health [17].

The initiation and progression of periodontitis are determined by the complex interplay between an altered subgingival microbiota and the host’s dysregulated local immune-inflammatory response. Periodontitis is defined by a reciprocal cause-and-effect relationship associated with the two pathogenetic players. The dysbiotic microbiota triggers inflammation and causes progressive periodontal tissue breakdown. In turn, persisting inflammation creates a favorable environment for the proliferation of certain pathogenic bacteria, further perpetuating periodontal inflammation, local tissue destruction, and the expansion of the pathogenic microbiota [17,22,23].

The modifications in the environmental conditions determined by inflammation may induce the outgrowth of pathobionts (commensal bacteria that, under disrupted homeostasis conditions, can provoke dysregulated inflammation and disease) beyond a threshold that can trigger periodontal destruction [17,24]. Consequently, bacteria associated with periodontitis or inflammophilic pathobionts outgrow selectively at the expense of those species that fail to adapt to the new environmental conditions and induce a dysbiotic imbalance in the subgingival microbiota [25,26].

The dysbiotic subgingival community acquires an increased ability to induce pro-inflammatory cytokines release by the host cells, setting off disproportionate immune-inflammatory reactions [27]. In periodontitis, the dysregulated host immune response is a consequence of either host immunoregulatory defects or its subversion by the dysbiotic microbiota that triggers inefficient local immune-inflammatory reactions in controlling bacterial outgrowth and pathogenicity [25].

Periodontitis-associated dysbiotic bacterial species need inflammation to sustain their nutritional needs. At the same time, bacteria assure self-protection by downregulating the host immune reactions, creating a non-inflammatory milieu unable to ensure nutrition for inflammophilic microorganisms. Some periodontopathogenic bacteria, including the keystone pathogen *Porphyromonas gingivalis,* overpass this paradox by manipulating the host response to uncouple only the bactericidal activity and no other inflammatory reactions, enhancing the adaptative fitness of the entire bacterial community [19].

Commensal bacteria can opportunistically trigger destructive inflammation as demonstrated by the association between periodontitis and uncultivable microbes such as *Filifactor alocis* and *Peptostreptococcus stomatis*, as well as other species from the genera *Prevotella*, *Megasphaera*, *Selenomonas*, and *Desulfobulbus* [28]. The recent analyses of some of these bacteria have identified virulence factors consistent with a pathobiont condition. For example, *Filifactor alocis* has the capacity to resist oxidative stress and to cause intense pro-inflammatory reactions [29].

Non-susceptible individuals tolerate the conversion of the symbiotic subgingival microorganisms’ community into a dysbiotic one due to their intrinsic biological phenotype [30]. The host-microorganism homeostasis can also be disturbed by congenital or acquired host immunoregulatory defects, systemic diseases, obesity, environmental factors, or aging [31].

The periodontitis-associated biofilm exhibits remarkable stability and resilience, making the spontaneous return to a health-related microbiota unlikely without any external intervention [32,33]. Disrupting subgingival biofilm through therapy disperses bacteria and triggers modifications in their gene expression, making them more vulnerable and more likely to be eliminated [17,34,35,36].

## 3. Goals and Limitations of Periodontal Therapy

The primary goal of periodontal treatment is to arrest periodontitis progression, preventing thus tooth loss and maintaining periodontal stability over time [37]. The desirable endpoints following the active periodontal therapy consist of achieving shallow periodontal pockets ≤ 4 mm, no 4 mm pockets with bleeding on probing, and a full-mouth bleeding score not exceeding 10% [38]. Finally, periodontitis treatment aims to rehabilitate oral functions complexly.

**Figure 1:**
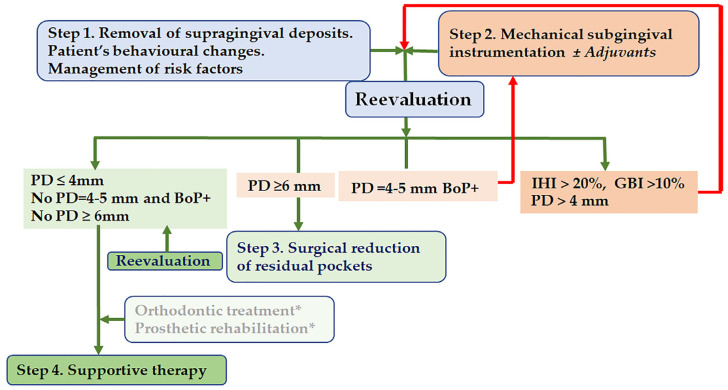
A brief overview of the periodontitis treatment protocol. Abbreviations: BoP = bleeding on probing, IHI = plaque index, PD = probing depth, * = applied in stage IV periodontitis.

The current EFP S3-level Practical Clinical Guideline elaborated for stage 1 to 3 periodontitis forms (Figure 1) recommends a basic treatment applicable for all periodontitis cases consisting of the supragingival plaque control plus the management of patients’ susceptibility and professional subgingival mechanical instrumentation that eliminates subgingival calculus and biofilm deposits [3]. The first and second-step therapies reduce dysbiosis and suppress local inflammation [6], improving clinical parameters.

If the desirable therapeutical endpoints have not been achieved at reevaluation, residual pockets should be managed using surgical pocket reduction approaches. After active periodontal therapy, regular supportive periodontal care through combined preventive and therapeutical approaches maintains periodontal stability over time [3].

However, an important shortcoming of the subgingival mechanical instrumentation is the impossibility of always eradicating all periodontopathogens, especially from inaccessible sites such as deep periodontal pockets, root concavities, and grooves or furcation lesions of multirooted teeth. Residuals favor microbial recolonization and reinfection [3,5,8,39].

Various adjunctive chemical or physical therapeutical agents (Table 1) have been promoted to improve the periodontal outcomes of subgingival mechanical instrumentation. Local antimicrobial agents have been extensively used as adjunctive agents in periodontitis therapy [10,40]. Still, for many of them, there is limited evidence concerning their effectiveness, so their recommendation level is rather inconsistent [3,9].

Systemic antibiotics have been generally recommended in severe forms of periodontitis [3,42] in order to target intratissular or mucosal colonizing periodontal pathogens and support the host’s immune deficiencies [43,44,45,46,47]. Adjunctive systemic antibiotics, particularly metronidazole, amoxicillin, or their combination, significantly improved clinical endpoint parameters and the microbiological profile at diseased sites compared to subgingival mechanical instrumentation alone [3,43,45,46,47,48]. However, the major concern related to the increased risk of bacterial resistance and other drawbacks associated with adjunctive systemic antibiotic therapy drastically limits their use in periodontitis patients [3,42,48,49].

Locally delivered antimicrobials are an alternative to overcome the shortcomings of systemic antibiotics. There is a large offer of topical adjunctive antimicrobials under various pharmaceutical forms, such as irrigants, stripes, films, fibers, gels, chips, microspheres, or nanoparticles that the clinician can directly deliver at the affected site [10,50,51]. Of interest in clinical practice are locally delivered slow-release antimicrobials, which maintain an adequate bioavailability over a more extended period [44,51,52], induce fewer side effects, and improve patient adherence [10,39,53,54]. Adjunctive locally delivered antimicrobials in combination with subgingival instrumentation can be effective in obtaining a significant reduction of the probing depths (WMD = 0.365 mm) and of the clinical attachment gains (WMD = 0.263 mm) after 6 to 9 months as compared to subgingival mechanical instrumentation alone [10]. The availability of efficient commercial products of this type is low, and the relative clinical effectiveness can be considered only for some products based on chlorhexidine (Periochip^®^—Dexcel Pharma Technologies Ltd., Jerusalem, Israel), minocycline (Arestin^®^—OraPharma, Bridgewater Township, NJ, USA) and doxycycline (Ligosan^®^—Heraeus Kulzer GmbH, Hanau, Germany; Atridox^®^ TOLMAR Inc., Fort Collins, CO, USA) [3,10].

## 4. The Pharmaceutical Formulation of a Sulphonic/Sulfuric Acids-Based Antimicrobial Desiccant Agent in Relation to Its Mechanisms of Action

### 4.1. HY Composition and Clinical Indications

Common oral antiseptics target the inhibition of plaque attachment or bacterial destruction through various mechanisms [44,50,55,56]. However, most of these products lack the potential to disorganize already-formed biofilms [57] through the destruction of the extracellular matrix [35,56,58], which contributes to the maintenance of a highly hydrated and protective environment for residing bacteria [34,59,60].

HY is a less studied and used therapeutical agent chemically interacting with the water from the biofilm matrix without carrying any of the side effects associated with common antimicrobials. HY is a desiccant agent formulated as a liquid or gel containing an aqueous mixture of hydroxybenzenesulfonic, hydroxymethoxybenzene, and sulfuric acids that can be used in conjunction with the mechanical subgingival instrumentation [13].

Due to its desiccation, cauterization, and antimicrobial properties, this product has been initially recommended in the symptomatic treatment of recurrent aphthous stomatitis [15]. The hydroxybenzenes have a keratolytic action and are combined with sulfonate groups and sulfuric acid. They have been proven to denature, precipitate, and coagulate tissue debris, creating a protective layer over ulcerated surfaces. Several studies reported significant alleviation of the pain sensation and improved healing after treating these oral lesions with HY [15,58,61]. More recently, HY has been promoted as a potential decontaminating agent suitable for periodontal and peri-implant therapy as well as for root canal treatment [16,62,63,64].

### 4.2. Denaturation Effect of HY

The concentrated mixture of sulfonic/sulfuric acid solution possesses a strong affinity for water, allowing the denaturation of the biofilms’ molecular structure by forming electrostatic bonds between the negative pole of oxygen atoms from the sulfate group and the positive pole of hydrogen atoms from water. This chemical interaction dehydrates the biofilm, shrinks the extracellular matrix, coagulates bacteria, and detaches the biofilm from the root and gingival surfaces [13,58,63,65]. The detachment of the deposits and the disruption of the subgingival biofilm through the action of HY makes dispersed bacteria vulnerable to environmental antimicrobial mechanisms [13,36,58,63].

Under the chemical desiccation and the physical disruption potential of sulfonate phenolics, in vitro, multispecies biofilms almost instantly detached from artificial tooth-like surfaces, as revealed by real-time dynamic imaging using a flow cell system integrated with a real-time cross-polarization optical coherence tomography system [57]. Another in vitro study examining through non-contact high-resolution images captured with a 3D Nonlinear Optical Microscope tooth samples covered with biofilm, placed in an artificial mouth model, and exposed to different chemical and physical treatment approaches reported that HY alone caused only a modest disruption of 17% of the biofilm [66]. The association of HY with mechanical dip air/water spray rising showed an almost complete biofilm removal, with less than 10% residual biofilm remaining on the enamel surfaces [66], emphasizing the importance of mechanical instrumentation for biofilm removal. Another experimental study found HY to be relatively ineffective in disrupting or removing a four-week-old *Enterococcus faecalis* biofilm as compared to 6% NaOCl [67].

### 4.3. Antimicrobial and Anti-Inflammatory Properties of HY

Some studies focused on the antimicrobial effects of HY. An in vitro study reported a strong and comparable inhibitory, bactericidal, and fungicidal activity for HY, chlorhexidine, and sodium hypochlorite, at relatively low concentrations, even in multi-drug-resistant microbial strains, including *Staphylococcus aureus*, *Enterococcus faecalis, and Candida glabrata*. The minimal inhibitory concentration of HY against bacteria was 0.39%, with a minimal bactericidal concentration ranging from 0.39% to 0.78%, while the minimal inhibitory concentration against yeasts ranged between 0.1% and 0.78%, and the minimal fungicidal concentration ranged from 0.78% to 1.6% [68]. Other in vitro studies also demonstrated a strong bactericidal action of HY against *Enterococcus fecalis,* even at a reduced concentration [69,70]. HY demonstrated a considerable bactericidal potential against *Enterococcus fecalis* biofilm, which was significantly increased as compared to 0.9% NaCl, SilverSol/H_2_O (*p* < 0.05), but inferior to 6% NaOCl [67].

Different clinical studies assessed HY antimicrobial activity against periodontal pathogens and the effects on periodontitis-associated inflammatory markers. A single HY irrigation in periodontitis patients determined a marked reduction in the levels of some periodontal pathogens (*Aggregatibacter actinomycetemcomitans*, *Porphyromonas gingivalis*, *Tannerella forsythia*, *Treponema denticola*, *Campylobacter rectus*, *Eubacterium nodatum*, *Fusobacterium nucleatum*, *Fusobacterium periodonticum*, *Peptostreptococcus (Micromonas) micros*, *Prevotella intermedia, Capnocytophaga* sp. (*gingivalis*, *ochracea*, *sputigena*), *Eikenella corrodens*) as compared to the baseline microbiological profile, while a second HY irrigation in conjunction with subgingival mechanical instrumentation further reduced bacterial DNA to a level below the DNA detection limit. The crevicular inflammatory mediators (MMP-8, IL-1β, IL-6, and TNF-α) could not be detected after the first HY application [71].

HY decontaminating potential in the treatment of peri-implantitis revealed that the use of HY alone or in combination with air abrasion with erythritol, amorphous silica, and 0.3% chlorhexidine powder mixture induced a significant reduction of the implant-associated biofilm viability (3.14 ± 0.21 log10(CFU/mL), 3.24 ± 0.24 log 10(CFU/mL)) as compared to implants not treated at all [(7.48 ± 0.12 log 10(CFU/mL)], *p* = 0.012 and *p* = 0.037, respectively [37]. A considerable reduction of the total bacterial load and the red complex bacteria was reported immediately after HY application in association with non-surgical or surgical treatment of peri-implantitis. However, the microbiological parameters tended to increase after three months following the initial care [72].

A randomized clinical study followed over three months the microbiological outcomes after different subgingival therapeutical regimens used for periodontitis treatment. HY used as monotherapy was equally effective as ultrasonic instrumentation in reducing the subgingival bacterial load. After six weeks, the combination of ultrasonic instrumentation and HY determined a superior reduction of the anaerobic bacterial load compared to the mechanical instrumentation alone (*p* = 0.018). However, at the three-month follow-up, after two repeated treatment sessions, there was no significant difference in the anaerobic bacterial burden between the group treated with HY and ultrasonic instrumentation and the group treated only with ultrasonic instrumentation [63].

Another split-mouth randomized clinical study reported that as compared to subgingival mechanical instrumentation alone, the association with HY significantly reduced the proportion of some bacteria of the orange complex (*Fusobacterium nucleatum*, *Fusobacterium polymporphum*, *Fusobacterium periodonticum*, *Prevotella intermedia*) (*p* < 0.001) and all bacteria of the red complex (*p* < 0.001), the levels of all investigated inflammatory mediators IL-1β (*p* < 0.05), IL-10 (*p* < 0.05) and TNF-α (*p* < 0.05) as well as of the IL-1β/IL-10 ratio (*p* < 0.001) from the gingival crevicular fluid after one year [65]. A significant reduction of the total subgingival bacterial load and the red complex periodontopathogens (*Porphyromonas gingivalis*, *Tannerella forsythia*, *Treoponema denticola*) has also been reported after a single subgingival HY application in periodontitis patients [61].

Based on the available preclinical and clinical studies, chemical cleansing could be a predictable method to remove biofilm and eliminate pathogens associated with periodontitis and peri-implant polymicrobial, heterogeneous infections [73], which may also enhance the clinical effects. Although HY seems to possess an overall favorable biofilm-disrupting potential and suitable antimicrobial properties, it cannot substitute mechanical instrumentation. Due to the high heterogeneity of the available studies in terms of study design, testing methods, and investigated outcomes, further research is required to support a definite conclusion regarding HY anti-infective efficacy.

## 5. Biocompatibility of a Sulphonic/Sulfuric Acids-Based Antimicrobial Desiccant Agent

The biocompatibility of local antimicrobial agents is an important property that must be considered in clinical practice, and yet it is still a topic of debate because of their close relationship with periodontal structures and possible negative influences on the reparative processes following the second-step therapy. Some investigations indicate that locally delivered chemical substances, including antibiotics, can have detrimental effects on the viable cells of periodontal tissues. For example, concentrations of ≥30 μM minocycline and ≥100 μM doxycycline substantially decreased human gingival and periodontal ligament fibroblasts [74]. A 0.5 mg/mL minocycline concentration negatively influenced osteoblast proliferation [75]. High levels of tetracycline, minocycline, and doxycycline determined a dose-dependent decrease in the differentiation potential of osteoprecursor cells [76]. Chlorhexidine induced a cytotoxic effect on human periodontal cells such as gingival fibroblasts, gingival epithelial cells, periodontal ligament cells, and osteoblasts [77,78,79,80,81]. Different concentrations of sodium hypochlorite had cytotoxic effects on human periodontal ligament cells [79] and negatively affected the viability and morphology of human bone marrow mesenchymal stem cells (MSCs) [82] as well impaired the viability and proliferation of gingival MSCs [14].

HY induces molecular denaturation of organic materials and tissue coagulation of the outermost layers of the superficial periodontium. However, the moderate denaturation reactions would not induce the destruction or acidification of oral tissues [63]. Clinicians have raised some concerns about the possible oral cytotoxicity of HY. Currently, there is very scarce information concerning HY biocompatibility, although HY passed the conventional cytotoxicity test before its approval for clinical use. The product’s influence on periodontal tissues is provided mainly by indirect data than by direct studies using periodontal-derived cell lines. For instance, HY used for direct pulp capping in a canine model determined better outcomes in terms of pulp vitality, presence of odontoblasts, and new dentine formation compared to a conventional calcium hydroxide product [83]. HY may have only a limited cytotoxic effect or even no cytotoxic effect on MG-63 osteoblast-like cells [37]. HY induced a dose-dependent inhibition of human gingival MSC proliferation but provided no significant negative influence on cells’ viability as quantified by the MTT test regardless of its concentrations [14].

Further cytotoxicity assays using human oral-derived MSCs are needed since standard tests cannot predict the in vivo biological impact of dental products, and animal cell lines are less sensitive than human cell lines [84,85]. Moreover, new studies may contribute to a better understanding of the biological effects of HY on different components of the periodontium.

## 6. The Effect of Sulphonic/Sulfuric Acids-Based Antimicrobial Desiccant Agent on the Dental Root Morphology

By removing soft and hard deposits from the root surfaces, the mechanical subgingival instrumentation creates a compatible environment for the colonization of a harmonious subgingival microbiota and an optimal development of local reparative processes at the tooth-periodontium interface [86]. However conservatively performed, the subgingival mechanical instrumentation induces morphological and surface roughness modifications of the roots. Cementum removal exposes the dentin and opens dentinal tubules, which can lead to post-operative dentinal hypersensitivity [87] and possible bacterial penetration [88,89,90,91]. Rough root surfaces support bacterial recolonization and calculus formation preventing at the same time the gingival fibrous tissue reattachment [89].

**Figure 2:**
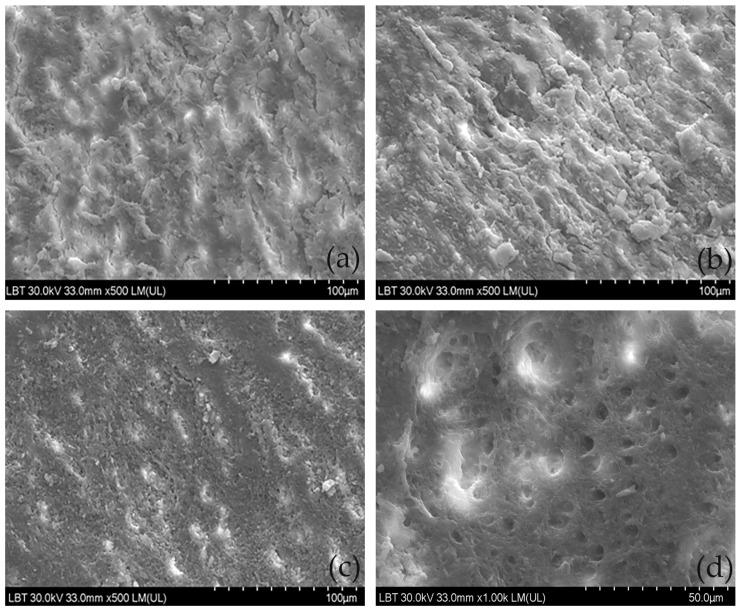
Root surface modifications on samples treated with subgingival mechanical instrumentation and subgingival mechanical instrumentation in conjunction with HY. (**a**) Brief curette scaling; (**b**) energic curette scaling, (**c**) ultrasonic scaling, (**d**) curette scaling, and HY application.

Subgingival mechanical instrumentation accumulates debris onto the root surfaces, also known as the smear layer (Figure 2a–c), which limits the spreading of specialized cells and impairs periodontal wound healing [86,92]. Several adjunctive subgingivally applied agents may enhance the removal of the smear layer and stimulate the colonization of specialized cells [77,93], thus creating the premises for the development of a new connective tissue attachment [86,92]. However, some products, such as citric acid, tetracycline, or EDTA, did not provide any additional clinical benefits in an attempt to regenerate an affected periodontium [94].

HY has been reported to dissolute the smear layer from root canals and root surfaces (Figure 2d) due to the demineralizing effects of sulfuric acids [69,95]. HY was almost as effective as 17% EDTA in the smear layer removal from the coronal and middle thirds of root canals during endodontic treatment. However, only a moderate amount of smear layer removal from the apical third of the root canal was reported for HY [69].

The SEM analyses showed that applications of HY onto instrumented roots coated by a continuous smear layer eliminated the smear layer more efficiently than sodium hypochlorite (Perisolv^®^—RLS Global AB, Mölndal, Sweden) or silver nanoparticles (Perioflush^®^—Dental Life Sciences, ARKONA Laboratorium Farmakologii Stomatologicznej, Nasutów, Poland), resulting in areas of partially opened dentin tubules of around 3–4 µm alternating with areas of continuous smooth, compact smear layer [14]. Root surfaces treated with HY seem to be more biocompatible than those treated with other products based on sodium hypochlorite (Perisolv^®^) and silver nanoparticles (Perioflush^®^), as seen in the elongated, well-attached gingival mesenchymal stem cells cultured on HY-treated samples [14].

## 7. Clinical Outcomes after the Use of the Sulphonic/Sulfuric Acids-Based Antimicrobial Desiccant Agent in Periodontitis Treatment

Because of its properties, HY was suggested to be a promising adjunctive product to be associated with subgingival mechanical instrumentation [13] in order to enhance periodontal clinical outcomes, especially in severe periodontitis cases. HY has been less extensively investigated in clinical studies as opposed to adjunctive antibiotic-based treatments [10,46]. Some studies evaluated different therapeutical HY regimens in the second or third-step therapy for periodontitis and in the treatment of periodontal abscess, reporting different primary and secondary outcomes (Table S1, see Supplementary Materials).

A case report study investigated HY decontamination efficacy in treating acute periodontal abscesses [58], which are relatively frequent among periodontitis patients [96]. In deep periodontal pockets ranging from 7 to 15 mm with localized purulent infection and mostly in association with infrabony defects, local decontamination with HY alone or in combination with flap surgery determined a marked reduction of the probing depths of up to a maximum of 4 mm and no bleeding on probing, in a relatively short period after treatment [58]. Another case report indicated a 2 mm periodontal pocket depth reduction at 15 days after subgingival application of HY in a first maxillary molar with acute periodontal abscess. Alleviation of the symptoms associated with inflammation occurred the following day after treatment [97].

The complete relief of clinical inflammatory signs was reported after subgingival administration of HY plus air abrasion with glycine powder, ultrasonic mechanical instrumentation, and photodynamic therapy in a seventy-year-old patient with rheumatoid arthritis [98]. However, no firm conclusions can be drawn on the utility of HY in periodontitis associated with rheumatoid arthritis cases, although the adjunctive use of the desiccant agent would be justified in this category of patients susceptible to developing more severe forms of periodontitis [98,99].

Three randomized controlled clinical trials reported the effects of HY on different periodontal parameters after its adjunctive use in periodontitis therapy. A pilot study with a split-mouth design reported a significantly greater reduction of the dental plaque and bleeding on probing scores as well as of the gingival indices in periodontitis patients after supra- and subgingival mechanical instrumentation combined with the topical administration of HY (combined therapy) as compared to those receiving only mechanical instrumentation. After three months, there were no considerable differences in the pocket depth reduction between sites treated with the combined therapy and those mechanically instrumented (4.69 ± 1.7 mm vs. 4.95 ± 1.7 mm, *p* = 0.04) [63]. On the contrary, a randomized split-mouth controlled trial reported a more important probing depth reduction (3.25 ± 0.57 mm vs. 2.23 ± 0.31 mm, *p* < 0.05) and clinical attachment gain (4.21 ± 0.34 mm vs. 3.16 ± 0.29 mm, *p* < 0.001) in patients treated with combined therapy as compared to subgingival mechanical instrumentation alone [65].

A more recent parallel, randomized clinical trial, in which multirooted teeth with deep periodontal pockets indicated for extraction were treated with combined therapy or only with subgingival mechanical instrumentation, evaluated on photographs the total area and the percentage of root surface covered with residual dental plaque and calculus as primary treatment outcomes. A significantly reduced root surface area (*p* < 0.001) was covered by residual plaque and calculus deposits in the group treated with combined treatment (5.17 ± 6.69 mm^2^ and 6.67 ± 8.72 mm^2^, respectively) as compared with sub-gingival mechanical instrumentation alone (15.96 ± 13.64 mm^2^ and 10.90 ± 7.69 mm^2^, respectively). Considering the percentages of soft and calcified deposits, a significantly reduced percentage of root surface covered by residual plaque (*p* < 0.001) was observed in the group treated with the combined approach (10.63 ± 12.79%) as compared to the group treated with the single approach (17.32 ± 10.68%). However, a significantly higher percentage of residual calculus (*p* < 0.001) was detected in the group treated with the combined therapy (46.90 ± 33.76%) as opposed to the other group (23.65 ± 18.27%). In addition, the mean cleaning depth was significantly lower (*p* < 0.001) for the teeth treated with the combined therapy (4.41 ± 2.96 mm) as compared to those treated by mechanical instrumentation alone (2.67 ± 2.70 mm). These results suggest that HY may enhance the efficacy of biofilm removal but does not bring any additional benefits to the calculus deposits removal in deep periodontal pockets of teeth with more complex morphology [100].

Secondary effects after HY applications have also been evaluated in these clinical studies. Thus, fewer patients reported pain during the second-step therapy or post-operative dentinal hypersensitivity when the combined therapy was applied as opposed to the subgingival mechanical instrumentation alone [63]. Moderate pain lasting no more than three days was reported by patients receiving subgingival administration of HY without anaesthesia for the treatment of periodontal abscesses. No persistent dental hypersensitivity was reported by these patients [58]. Topical HY applied in the abscess-associated periodontal pockets induced marked recessions of 3 to 5 mm due to post-therapeutical healing [58]. The full-mouth administration of HY extended by about 5 min the duration of the treatment session, which did not considerably affect the total treatment time in the group treated with the combined therapy compared to the control group [63].

## 8. Clinical Outcomes after the Use of the Sulphonic/Sulfuric Acids-Based Antimicrobial Desiccant Agent in Peri-Implantitis

HY has been promoted as a potential therapeutical agent to manage peri-implantitis (Table S1) since no firm treatment approaches are proposed for this clinical condition [101]. Three case report studies treating mild peri-implantitis with locally delivered HY as a single non-surgical therapy [72,102] or in association with flap surgery and guided bone regeneration [72,103] indicated an up to 4 mm reduction of the probing depths [72,102], the complete absence of bleeding on probing [102], physiological probing depths and more than 90% radiographic bone fill of the bone defects [103]. Another case report of generalized periodontitis and peri-implantitis showed that the complex treatment consisting of flap surgery, decontamination with HY, and guided bone regeneration determined normal probing depths around the affected teeth and implant [97]. However, it is difficult to isolate the impact of HY on the reported treatment outcomes.

A one-year follow-up clinical study assessing patients with severe peri-implantitis, treated with open flap debridement and one-time decontamination with HY, reported an obvious improvement of the peri-implant status as revealed by the mean probing depth reduction of 3.1 mm, a mean attachment gain of 3.6 mm, and a mean bone fill of 58.6% [64].

The main limitations of some of the cited clinical studies, mainly case reports, are the small number of recruited patients, the lack of comparison groups, the risk of selection bias, the lack of standardized assessment protocols, and the inability to generalize the results. Thus, further randomized controlled clinical studies are needed to assess the clinical efficacy of HY as part of periodontal and peri-implant therapy.

## 9. Conclusions

Due to its proven antibacterial properties, HY may be a promising therapeutical agent for the non-surgical and surgical treatment of periodontitis, peri-implantitis, endodontic infections, and even mucosal ulcers.

The adjunctive use of HY can bring additional clinical benefits, reduce the need for invasive therapeutical procedures and enhance the healing of the tissues in treating both periodontal and peri-implant lesions. Moreover, HY has the potential to partially remove the smear layer from root surfaces more efficiently than other locally adjunctive antimicrobial agents, which can facilitate, to some extent, periodontal tissue reattachment at the intervention sites.

Nevertheless, more in vitro studies with different designs and larger sample sizes are needed to precisely determine the influence of the sulfonic/sulfuric acid-based decontaminating agent on the root surfaces and cell adhesion and to provide additional information on the biocompatibility of this product. Moreover, long-term clinical studies with larger samples of patients are needed to determine the product’s effectiveness in humans.

## Figures and Tables

**Table 1:** Clinical Practice Guideline recommendations of adjunctive therapies in second-step therapy [3] based on the GRADE system [41].

Adjunctive Approaches	Type	Recommendations for Clinical Practice
**Physical agents**	Lasers	Suggestion not to be used (GRADE B↓)
	Photodynamic therapy	Suggestion not to be used (GRADE B↓)
**Antimicrobials**	Sustained-release chlorhexidine	May be considered (GRADE 0↔)
	Locally administered antibiotics	May be considered (GRADE 0↔)
	Systemic antibiotics	Routine use is not recommended (GRADE A↓↓) May be considered for specific patient categories (GRADE 0↔)
**Hostmodulating** **agents**	Local statins	Recommendation not to be used
	Probiotics	Suggestion not to be used (GRADE B↓)
	Systemically administered sub-antimicrobial dose doxycycline	Suggestion not to be used (GRADE B↓)
	Systemic/local non-steroidal anti-inflammatory drugs	Recommendation not to be used (GRADE A↓↓)
	Omega-3 polyunsaturated fatty acids	Recommendation not to be used (GRADE A↓↓)

## Data Availability

Not applicable.

## Supplementary Materials

The following supporting information can be downloaded at: https://www.mdpi.com/article/10.3390/antibiotics12030456/s1, Table S1: Summarization of the clinical studies assessing periodontal and peri-implant treatment outcomes following the topical administration of HybenX.
