# Supplementary material for: A Local Desiccant Antimicrobial Agent as an Alternative to Adjunctive Antibiotics in the Treatment of Periodontitis: A Narrative Review
Source: Antibiotics (Basel). 2023 Feb 24;12(3):456. doi: 10.3390/antibiotics12030456 (PMC10044681; doi:10.3390/antibiotics12030456)
Supplement: Supplementary file 1 [file antibiotics-12-00456-s001.zip › antibiotics-2201065-supplementary.pdf]

Table S1. Summarization of the clinical studies assessing periodontal and peri-implant treatment outcomes following the topical administration of HybenX.

Author Year Reference Number	Study Design	Clinical Cases	Objectives	Therapeutical Intervention	Main treatment outcomes
Pini-Prato et. al., 2016 [58]	Case report	Cases of acute periodontal abscess	Assessment of the clinical treatment outcomes	HY subgingival administration alone or in combination with flap surgery No systemic antibiotic therapy	Complete resolution of the acute periodontal abscess after treatment<br>Considerable reduction of the initial PD, ranging from 7 to 15 mm to PD ranging from 1.5 to 4mm, without BOP recorded at the variable follow-up intervals after treatment<br>Marked increase of the initial GR ranging from 3 to 5 mm for most patients
Mancini et al., 2016 [97]	Case report	A case of acute periodontal abscess / A case of generalized periodontitis and peri-implantitis	Assessment of the clinical treatment outcomes	HY subgingival administration without subgingival mechanical instrumentation or systemic antibiotic therapy<br><br>HY topical administration with flap surgery and guided bone regeneration<br>No systemic antibiotic therapy	At 15 days follow-up:<br>Complete resolution of the acute periodontal abscess<br>Reduction of the initial PD from 7.5 mm to 5.5 mm and of the initial CAL of 5.5 mm to 5 mm<br><br>At 4 months follow-up:<br>Reduction of the initial PD from 6 mm to 1.5 mm and of the initial CAL of 4 mm to 2 mm at the mesial surface of the first molar<br>Reduction of the initial PD from 8 mm to 3 mm and of the initial CAL of 6 mm to 3 mm at the mesial surface of the implant
Nardi et al., 2013 [98]	Case report	A case of rheumatoid arthritis patient with periodontitis and peri-implantitis	Assessment of the clinical treatment outcomes	Individualized oral hygiene routine instructions<br>Professional oral hygiene regime consisting of air-abrasion with glycine powder, HY subgingival administration, ultrasonic and manual subgingival mechanical instrumentation, photodynamic therapy	At 1 month follow-up:<br>No detectable clinical signs of periodontal / peri-implant inflammation<br>No detailed description with respect to the clinical periodontal / peri-implant parameters

Lauritano et al., 2015 [61]	Case series	Patients with moderate periodontitis	Assessment of microbiological treatment outcomes	One-time HY subgingival administration	At 15 days follow-up:<br>A marked reduction of *Porphyromonas gingivalis, Tannerella forsythia, Treoponema denticola* and of the total subgingival bacterial load
Lombardo et al., 2015 [63]	RCT	Patients with mild and severe periodontitis	Assessment of clinical periodontal and microbiological treatment outcomes	Test group: HY subgingival administration + ultrasonic subgingival mechanical instrumentation<br>vs.<br>Control group: ultrasonic subgingival mechanical instrumentation	At 3 months follow-up:<br>A significant improvement in the plaque, BOP and gingival index scores in the test group compared to the control group<br>No significant difference in PD and CAL between the two groups<br>No significant difference in the bacterial load reduction between the two groups
Isola et al., 2018 [65]	RCT	Patients with periodontitis	Assessment of clinical periodontal, immunological, and microbiological treatment outcomes	Test group: HY subgingival administration + manual and ultrasonic subgingival instrumentation<br>vs.<br>Control group: manual and ultrasonic subgingival mechanical instrumentation	At 12 months follow-up:<br>Significantly improved PD, CAL, and BOP in the test group compared to the control group<br>Significant reduction of Porphyromonas gingivalis, Tannerella forsythia, Treponema denticola, Fusobecterium nucleatum, Fusobecterium polymporphum, Fusobecterium periodonticum, Prevotella intermedia in the test group compared to the control group<br>Significant reduction of the mean level of IL-1β, TNF-α, IL-1β/IL-10 from the gingival crevicular fluid in the test group compared to the control group
Zafar et al., 2021 [100]	Experimental RCT	Patients with stage III/IV periodontitis and minimum one hopeless	Assessment of residual subgingival plaque and calculus deposits and of the mean cleaning depth in deep periodontal pockets	Test group: HY subgingival administration + manual and ultrasonic subgingival mechanical instrumentation<br>vs.<br>Control group: manual and ultrasonic subgingival mechanical instrumentation<br><br>Subsequent extraction of a hopeless posterior multirooted teeth	Immediately following treatment and extraction of the hopeless teeth:<br>A significantly narrower subgingival root area covered by residual plaque and calculus deposits in the test group compared to the control group<br>A significantly lower percentage of residual plaque in the test group compared to the control group<br>A significantly lower percentage of residual calculus deposits in the test group compared to the control group<br>A significantly higher mean cleaning depth for the control group compared to the test group

Lopez et al., 2016 [72]	Case report study	Cases of peri-implantitis	Assessment of clinical peri-implant and microbiological treatment outcomes	HY topical administration alone or in combination with open flap debridement and guided bone regeneration	At 3 months follow-up:<br>Marked reduction of the red complex bacteria and of the periodontal pockets in milder forms of peri-implantitis<br>Lack of suppuration but a tendency of increase in the total bacterial load and in the count of red complex bacteria
Pini-Prato et al., 2016 [102]	Case report study	Cases of peri-implantitis	Assessment of the clinical and radiographic treatment outcomes	HY topical administration without mechanical instrumentation or systemic antibiotic therapy	Considerable reduction of the initial PD, ranging from 5 to 8mm, to PD of up to 4mm, without BOP and a modest increase of the GR at the 3 months follow-up.<br>Healthy peri-implant soft tissues at the 6 months follow-up<br>Signs of bone remineralization at the 6 months follow-up for most patients<br>Treatment results remained stable at 6 months follow-up
Lombardo et al., 2015 [103]	Case report study	A case of peri-implantitis	Assessment of the clinical and radiographic treatment outcomes	Open flap debridement with HY topical administration, low abrasive air powder abrasion and guided bone regeneration	At 2 years follow-up:<br>Reduction of the mean PD of up to 5.5 mm; CAL of up to 5.1 mm complete absence of BOP<br>Increase of radiographic first bone-to-implant contact of up to 8 mm<br>Percentage of radiographic bone fill of more than 90%
Lopez et al., 2021 [64]	Clinical observational study	Patients with peri-implantitis	Assessment of the clinical and radiographic treatment outcomes	HY topical administration in combination with open flap debridement	At 12 month follow-up:<br>No suppuration in any of the cases<br>Mean PD reduction of 3.1 mm and mean attachment gain of 3.6 mm<br>BOP present in 6% of implant sites<br>Mean bone gain of 3.4 mm and mean percentage of radiographic bone fill of 58.6%

Abbreviations: BOP=Bleeding on probing; CAL=Clinical Attachment Level; GR=Gingival Recession, HY=HybenX®; IL=Interleukin, RCT=Randomized Controlled Trial; TNF=Tumor necrosis factor.
